# The effect of ginsenoside Rg3 combined with chemotherapy on immune function in non-small cell lung cancer: A systematic review and meta-analysis of randomized controlled trials

**DOI:** 10.1097/MD.0000000000033463

**Published:** 2022-04-07

**Authors:** Song Gao, Cancan Fang, Tiancheng Wang, Wenjie Lu, Nan Wang, Lingfeng Sun, Wenyou Fang, Yang Chen, Rongfeng Hu

**Affiliations:** a Anhui Province Key Laboratory of Pharmaceutical Preparation Technology and Application; Anhui University of Chinese Medicine, Hefei, Anhui, China; b MOE-Anhui Joint Collaborative Innovation Center for Quality Improvement of Anhui Genuine Chinese Medicinal Materials; Key Laboratory of Xin’an Medicine (Anhui University of Chinese Medicine), The Ministry of Education, Anhui Province Key Laboratory of Chinese Medicinal Formula; Plant Active Peptide Function Food Innovative Manufacturing Industry Innovation Team, Hefei, Anhui, China; c School of Pharmacy, China Pharmaceutical University, Nanjing, Jiangsu, China; d Oncology Department of Integrated Traditional Chinese and Western Medicine, The First Affiliated Hospital of Anhui Medical University, Hefei, China.

**Keywords:** chemotherapy, ginsenoside Rg3, immune function, meta-analysis, non-small cell lung cancer

## Abstract

The occurrence and development of non-small cell lung cancer (NSCLC) are closely related to the immune status of the tumor-host. The immunosuppression caused by tumor cells and toxic side effects produced by chemotherapeutic drugs results in a decrease in immune function, ultimately leading to the failure of clinical chemotherapy treatment. Ginsenoside Rg3 has been clinically reported to have positive effects in enhancing immune function in patients. Thus, we screened and evaluated the quality of the evidence regarding the benefits of ginsenoside Rg3 and conducted a meta-analysis to assess the impact on improving immune function in NSCLC.

**Methods:**

The PubMed, EMBASE, Cochrane Library, CNKI, Weipu (VIP), and Wanfang databases were searched in this study, all from the time of library construction to January 2023.

**Results:**

In total,12 trials with a sample size of 1008 cases were included based on the eligible criteria. The results showed that compared with first-line chemotherapy alone, the combination of ginsenoside Rg3 and first-line chemotherapy could better improve level of the CD3^+^ T lymphocytes [mean difference (MD) = 4.72; 95% confidence intervals (CI): 3.92, 5.53; *P* < .00001], CD4^+^ T lymphocytes (MD = 4.93; 95% CI: 4.61, 5.26; *P* < .00001), CD8^+^ T lymphocytes (MD = 2.67; 95% CI: 0.93, 4.37; *P* = .003), CD4^+^/CD8^+^ T lymphocytes (MD = 0.20; 95% CI: 0.09, 0.32; *P* = .0006), increase the activity of nature killer cells (MD = 2.11; 95% CI: 0.58, 3.63; *P* = .007), recover the decline of the white blood cell count induced by chemotherapy, and improve the clinical efficacy for patients.

**Conclusion:**

This study confirmed that ginsenoside Rg3 has some efficacy advantages for improving immune function in patients with NSCLC.

## 1. Introduction

Lung cancer is the most deadly malignancy worldwide, with an estimated 1.8 million deaths from the disease in 2020, accounting for 18% of all cancer deaths.^[1]^ Approximately 85% of patients have an identical histological subtype, collectively known as non-small cell lung cancer (NSCLC), with lung adenocarcinoma and lung squamous cell carcinoma being the most common subtypes.^[2]^ Chemotherapy is currently the mainstay of treatment and is of great clinical value for those who cannot tolerate surgery and for adjuvant therapy after surgery.^[3]^ Platinum, paclitaxel, and docetaxel have become the first-line clinical chemotherapeutic agents. Although they have specific efficacy, they still face challenges such as uncertain efficacy and severe adverse reactions.^[4]^ Scientific studies have confirmed that the occurrence and development of tumors are closely related to the tumor-host immune status.^[5]^ Due to excessive proliferation of various immunosuppressive cells and overproduction of Th2-type cytokines, severe immunosuppression occurs at the tumor site, leading to the decreased immune response of T lymphocytes and natural killer cell (NK) cells.^[6]^ At the same time, the toxic side effects of chemotherapy, such as the decline of WBC, will further reduce the immune function of patients, which is one of the fundamental reasons for the failure of a variety of antitumor drugs.^[7]^

Traditional Chinese medicine has shown unique advantages in antitumor and immune regulation in recent years. Shenyi capsule, the main component of ginsenoside Rg3, is the first-class A anticancer drug of Chinese medicine monomer in China, which is mainly used in the adjuvant treatment of NSCLC.^[8]^ It is one of the ingredients extracted from Ginseng, which has the effect of cultivating vital energy and tonifying the qi and blood. Studies showed that ginsenoside Rg3 could effectively inhibit the development and metastasis of tumor cells by inducing tumor apoptosis, blocking Angiogenesis, inhibiting the epithelial transformation, and promoting reprogramming of M_2_-type tumor-associated macrophages.^[9–12]^ In combination with chemotherapeutic agents, ginsenoside Rg3 can remodel the immune microenvironment, restore the function of T lymphocytes, and relieve immunosuppression status, thus achieving synergistic multi-target therapy and improving the antitumor efficacy of chemotherapeutic agents.^[13]^ There are few previous studies on ginsenoside Rg3 in enhancing the immune microenvironment of cancer patients. Therefore, it is necessary to systematically evaluate the effect of ginsenoside Rg3 combined with chemotherapy on the immune function of patients with NSCLC to provide evidence-based evidence for clinical application.

## 2. Material and Methods

This study was conducted in accordance with the preferred reporting project (PRISMA) statement of systematic reviews and meta-analyses and was designed following previous similar publications.^[14–16]^ The protocol of this review was registered in the International Prospective Register of Systematic Reviews, and the trial registration number was CRD42023399449 (https://www.crd.york.ac.uk/PROSPERO). Ethical approval and consent of patient were not applicable for our meta-analysis, because we just included published literature.

### 2.1. Search strategy

The study was searched from PubMed, EMBASE, Cochrane Library, CNKI, Wanfang, and VIP databases, all of which were implemented using MeSH and free words. The investigations lasted from the established time until January 2023. All studies were searched, regardless of publication type and language. The 2 authors (Song Gao and Cancan Fang) independently searched all relevant studies in both English and Chinese databases using the following search strategy. For the Chinese databases, the following terms were used in combination: [Ginsenoside Rg3 or Rg3 or Shenyi capsule] AND [Lung cancer or non-small cell lung cancer]; For the English databases, the following terms were used in combination: [Ginsenoside Rg3 or Rg3 or Shenyi capsule] AND [Lung cancer or non-small cell lung cancer or NSCLC or Non-small cell carcinoma].

### 2.2. Inclusion and exclusion criteria

Inclusion criteria were as follows: Patients: patients with a confirmed diagnosis of NSCLC, regardless of age, sex, race, or clinical stage; Study design: randomized clinical controlled trial, regardless of language; Intervention: control group: conventional chemotherapy, test group: the same conventional chemotherapy regimen combined with ginsenoside Rg3; Outcome: at least 1 immune index was included.

Exclusion criteria were as follows: Duplicate publications; Nonclinical experimental studies (e.g., case reports, empirical summaries, animal and pharmacology-based trials); Studies in non-NSCLC patients; Studies not treated with chemotherapy; Studies without tumor-related immune indicators.

### 2.3. Data extraction

Two researchers (Song Gao and Can Can Fang) independently extracted information from the literature according to the inclusion criteria. In case of disagreement, the third researcher solved the problem through discussion. The contents of literature extraction were mainly included as follows: author, publication year, sample size, age, gender, NSCLC stage, intervention measures, and treatment cycle. The corresponding outcomes included the level of Peripheral blood T lymphocyte subsets (CD3^+^, CD4^+^, CD8^+^, CD4^+^/CD8^+^), the activity of NK cells, the rate of leukocytopenia, and the clinical efficacy.

### 2.4. Quality assessment

All literature data were input into the system review management software RevMan 5.3 (Cochrane Collaboration, Copenhagen, Denmark). The “Risk of Bias assessment” tool in the Cochrane manual was used for evaluation as follows: Random sequence generation; Allocation concealment; Blinding of participants and personnel; Blinding of outcome assessment; Incomplete outcome data; Selective reporting; Other sources of bias. The risk of bias was classified as “high, “unclear, or “low”.^[17,18]^

### 2.5. Statistical analysis

The statistical analysis was performed by using RevMan 5.3 and STATA 16.0 software (StataCorp, College Station, TX). Odds ratio with 95% confidence intervals (CI) was used as efficacy analysis statistics for dichotomous variables, and mean difference (MD) with 95% CI was used as efficacy statistics for continuous variables. A fixed-effects model was used when the results of the heterogeneity test of the studies were small (*P* > .10, *I*^2^ < 50%), and the random-effects model was used if there was significant statistical heterogeneity (*P* ≤ .10, *I*^2^ > 50%). Subgroup analysis was performed on the relevant outcomes based on the treatment cycle, and sensitivity analysis was performed to illustrate the impact of changing the study model on the pooled analysis.

## 3. Results

### 3.1. Study selection

**Figure 1.**
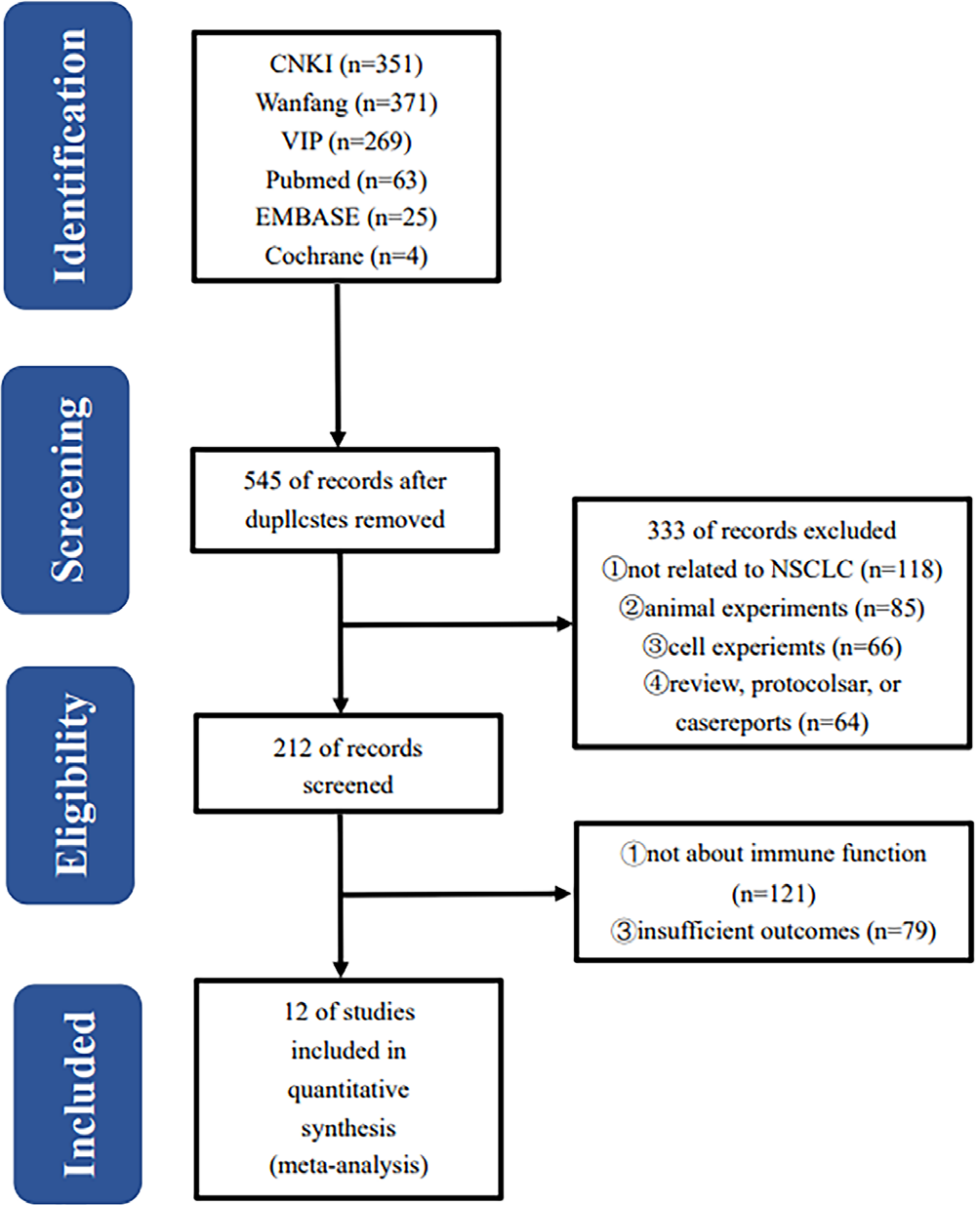
Flow chart of study selection process.

A total of 1083 relevant articles were initially retrieved, and 545 were left after eliminating duplicate reports by Note Express. Experimental studies of animal and pharmacological experiments or clinical studies unrelated to NSCLC were excluded after reading the title and abstract. The remaining 212 pieces of literature were read in total, and 12 were finally included when individual studies, incomplete experimental data, no control group, and no immune-related studies were excluded (Fig. 1).

### 3.2. Characteristics of the studies

As shown in Table 1, 12^[19–30]^ randomized controlled trials (RCTs) published between 2002 and 2023 were included in this study, with a total sample number of 1008 patients, all of whom were NSCLC patients aged 18 to 79. There was no statistical difference in the results of random allocation, and all the RCTs were comparable. Five hundred fifty-three patients in the experimental group received ginsenoside Rg3 orally plus chemotherapy, while 455 patients in the control group received chemotherapy only. The tumor stage was from Ib to IV, and the treatment period was 6 to 16 weeks. The included studies were examined for immune function at the end of treatment, and measured data were used to report the level of T lymphocyte subsets (CD3^+^, CD4^+^, CD8^+^, CD4^+^/CD8^+^), NK cell activity, the rate of leukocytopenia, and clinical efficacy, respectively.

**Table 1:** Characteristics for inclusion studies.

Study	Year	Sample size (E/C)	Age	Sex (M/F)	Stage	Experlment group (E)	Control group (C)	Treatment cycle	Pathological type
Yu et al^[19]^	2018	57/45	63.85	54/48	IIIb, IV	Rg3 20mg po.bid + C	GP	8 wk	CD3^+^; CD4^+^; CD8^+^; CD4^+^/CD8^+^; WBC; clinically efficacy
Hou et al^[20]^	2013	25/24	18–75	-	IIIb, IV	Rg3 20mg po.bid + C	C	6 wk	CD4^+^/CD8^+^; clinically efficacy
Liu and Zhou et al^[21]^	2007	35/35	56	43/27	IIIb, IV	Rg3 20mg po.bid + C	NP	6 wk	CD4^+^/CD8^+^; NK; clinically efficacy
Liu et al^[22]^	2007	35/33	69/70	47/21	IIIb, IV	Rg3 20mg po.bid + C	NP	6 wk	CD3^+^; CD4^+^; CD8^+^; CD4^+^/CD8^+^; WBC; clinically efficacy
Shi et al^[23]^	2018	31/31	69.67/68.34	41/21	Ib–IIIa	Rg3 20mg po.bid + C	PC	16 wk	CD3^+^; CD4^+^; CD8^+^; CD4^+^/CD8^+^
Shi et al^[24]^	2015	30/30	60.5/62.4	33/27	IIIb, IV	Rg3 20mg po.bid + Radiotherapy + C	Radiotherapy + DDP/CBP	12 wk	CD3^+^; CD4^+^; CD8^+^; CD4^+^/CD8^+^; clinically efficacy
Kang et al^[25]^	2020	34/34	64.5/64.1	36/32	IIIb, IV	Rg3 20mg po.bid + C	HP	6 wk	CD3^+^; CD4^+^; CD8^+^; CD4^+^/CD8^+^; clinically efficacy
Zhang et al^[26]^	2020	40/40	45.23/47.13	53/27	IIIa, IIIb, IV	Rg3 20mg po.bid + C	GP	16 wk	CD3^+^; CD4^+^; CD8^+^; CD4^+^/CD8^+^; WBC; clinically efficacy
Lin et al^[27]^	2002	115/31	53.8	109/42	II–IV	Rg3 20–30mg po.bid + C	EP/MVP	6 wk	CD3^+^; CD4^+^; CD8^+^; CD4^+^/CD8^+^; clinically efficacy
Pan et al^[28]^	2019	103/104	60.6/62.5	104/103	III–IV	Rg3 20mg po.bid + C	TP	9 wk	CD3^+^; CD4^+^; CD8^+^; clinically efficacy
Wang et al^[29]^	2015	28/28	42–75	37/23	Ib–IIIa	Rg3 20mg po.bid + C	CP	8 wk	CD3^+^; CD4^+^; CD8^+^; CD4^+^/CD8^+^; NK
Jin et al^[30]^	2011	20/20	70–78/70–79	24/16	II, IIIa	Rg3 20mg po.bid + C	GP	8 wk	CD4^+^/CD8^+^; NK; WBC

C = control group, CP = 500mg/m^2^ Pemetrexed + 25mg/m^2^ Cisplatin, DDP = Cisplatin, E = experimental group, EP = 40mg/m^2^ Etoposide + 30mg/m^2^ Cisplatin, GP = 1000mg/m^2^ Gemcitabine + 25mg/(d·m^2^) Cisplatin, HP = 10mg/d Hydroxycamptothecin + 25mg/d Cisplatin, MVP = 6-8mg/m^2^ Mitomycin + 3mg/m^2^ Vincristine + 30mg/m^2^ Cisplatin, NK = natural killer cell, NP = 25mg/m^2^ Vinorelbine + 25mg/m^2^ Cisplatin, PC = 135–175mg/m^2^ Paclitaxel + AUC 6.0–7.0 Carboplatin, PTX = Paclitaxel, TP = 75mg/m^2^ Paclitaxel + 25mg/m^2^ Cisplatin.

### 3.3. Quality evaluation of the studies

**Figure 2.**
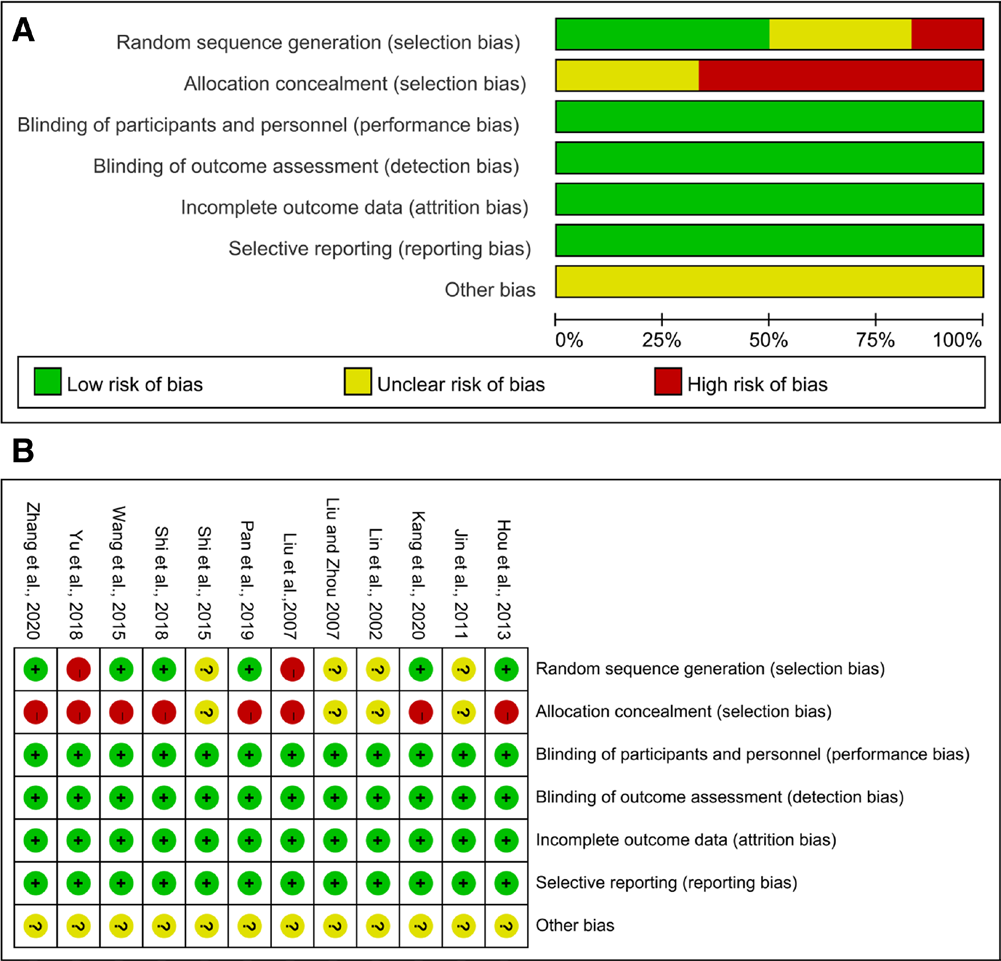
Risk of bias graph (A) and (B). Green refers to low risk of bias; yellow refers to unclear risk of bias; red refers to high risk of bias.

Among the 12 studies included, 5^[23,25,26,28,29]^ studies used a random number table for randomization, 1^[20]^ study used unequal random sequences for randomization, 1^[19]^ study randomized according to patient’s wishes, 1^[22]^ study randomized according to patients even-odd days at admission, and 4^[21,24,27,30]^ studies randomization method not specified. The Cochrane Handbook was used to evaluate the quality of the included studies, and the results were shown in Figure 2.

### 3.4. Findings from the meta-analysis

#### 3.4.1. CD3^+^ T lymphocytes.

**Figure 3.**
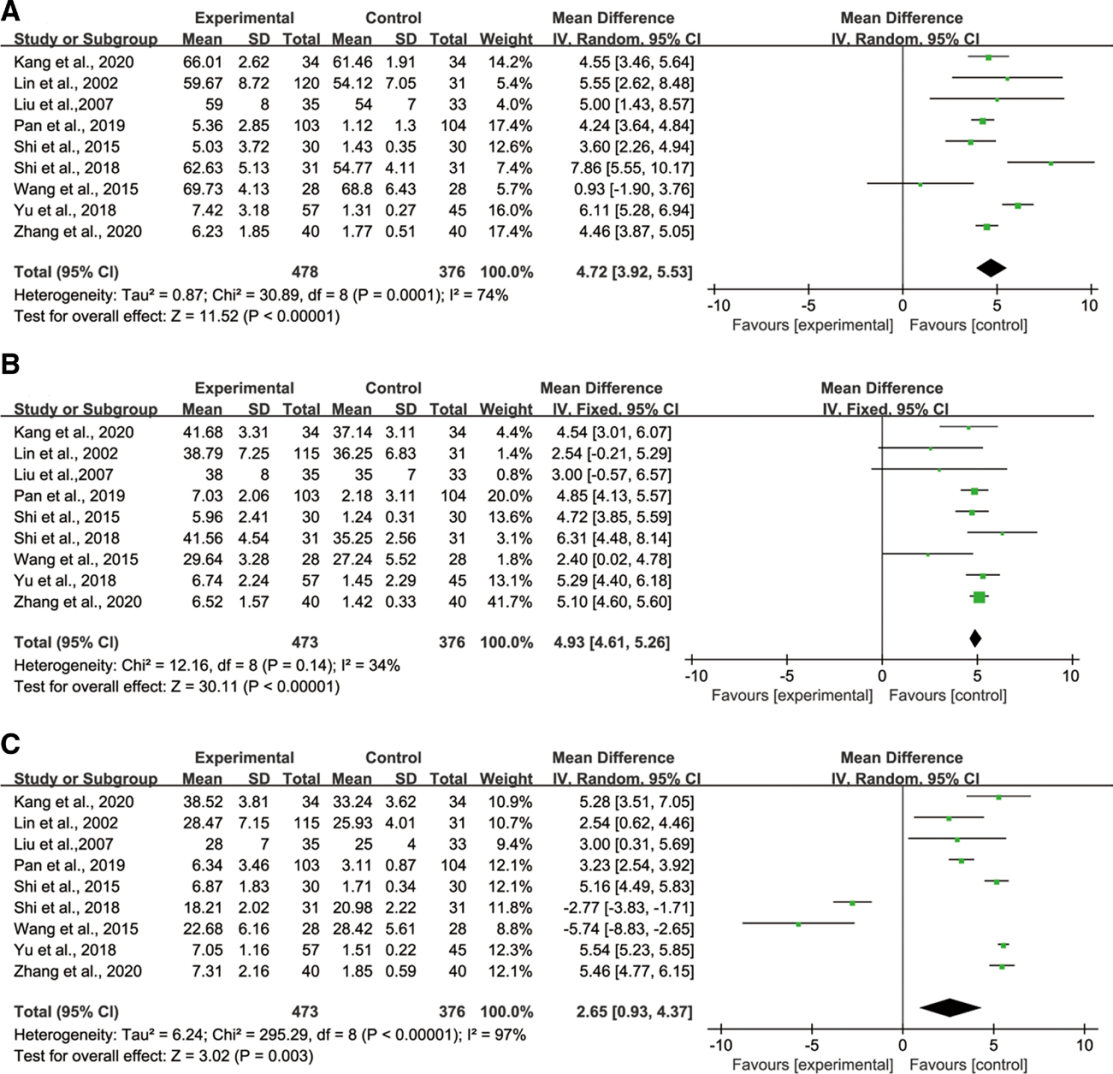
Forest plot (A) CD3^+^ T lymphocytes in Peripheral Blood, (B) CD4^+^ T lymphocytes in Peripheral Blood, and (C) CD8^+^ T lymphocytes in Peripheral Blood.

Nine studies reported the levels of CD3^+^ T lymphocytes in peripheral blood of NSCLC patients after administration of ginsenoside Rg3 combined with chemotherapy, including 478 patients in the experimental group and 376 patients in the control group (chemotherapy only). There was significant heterogeneity among studies (*P* = .0001, *I*^2^ = 74%), and Meta-analysis was performed using a random-effects model. Figure 3A showed that CD3^+^ levels were increased in the ginsenoside Rg3 combined chemotherapy group compared with the chemotherapy group, with a statistically significant difference (MD = 4.72; 95% CI:3.92, 5.53; *P* < .00001). Subgroup analysis was performed according to treatment cycle; administration time ≤ 8 weeks (MD = 5.30; 95% CI: 4.68, 5.92; *P* < .00001) was more effective in increasing the percentage of CD3^+^T cells in peripheral blood compared with administration time > 8 weeks (MD = 4.39; 95% CI: 3.99, 4.79; *P* < .00001) (Figure S1, Supplemental Digital Content, http://links.lww.com/MD/I758).

#### 3.4.2. CD4^+^ T lymphocytes.

Nine included RCTs measured CD4^+^ levels in peripheral blood after administration of ginsenoside Rg3 combined with chemotherapy and conventional chemotherapy, with 473 patients in the experimental group and 376 patients in the control group. The results of the heterogeneity test (*P* = .14, *I*^2^ = 34%) are shown in Figure 3B, and a fixed-effect model was used for Meta-analysis. The results showed that the CD4^+^ T cell count in the experimental group was higher than that in the control group, which could effectively increase the level of CD4^+^ T lymphocytes with a statistically significant difference (MD = 4.93; 95% CI:4.61, 5.26; *P* < .00001).

#### 3.4.3. CD8^+^ T lymphocytes.

The levels of CD8^+^ T lymphocytes in the ginsenoside Rg3 combined with the chemotherapy group and conventional chemotherapy group were studied in 9 RCTs, including 473 patients in the experimental group and 376 patients in the control group. There was heterogeneity among studies, and the random effect model could be used in meta-analysis. As shown in Figure 3C, the ginsenoside Rg3-chemotherapy group could improve the CD8^+^ T cell counts more considerably than the chemotherapy group (MD = 2.67; 95% CI: 0.93, 4.37; *P* = .003). Subgroup analysis confirmed that the level of CD8^+^ T lymphocytes could be improved more effectively when the treatment cycle was less than 8 weeks (MD = 5.33; 95% CI: 5.03, 5.62; *P* < .00001) than that was more than 8 weeks (MD = 3.72; 95% CI: 3.35, 4.09; *P* < .00001) (Figure S2, Supplemental Digital Content, http://links.lww.com/MD/I759).

#### 3.4.4. Raito of CD4^+^/CD8^+^ T lymphocytes.

**Figure 4.**
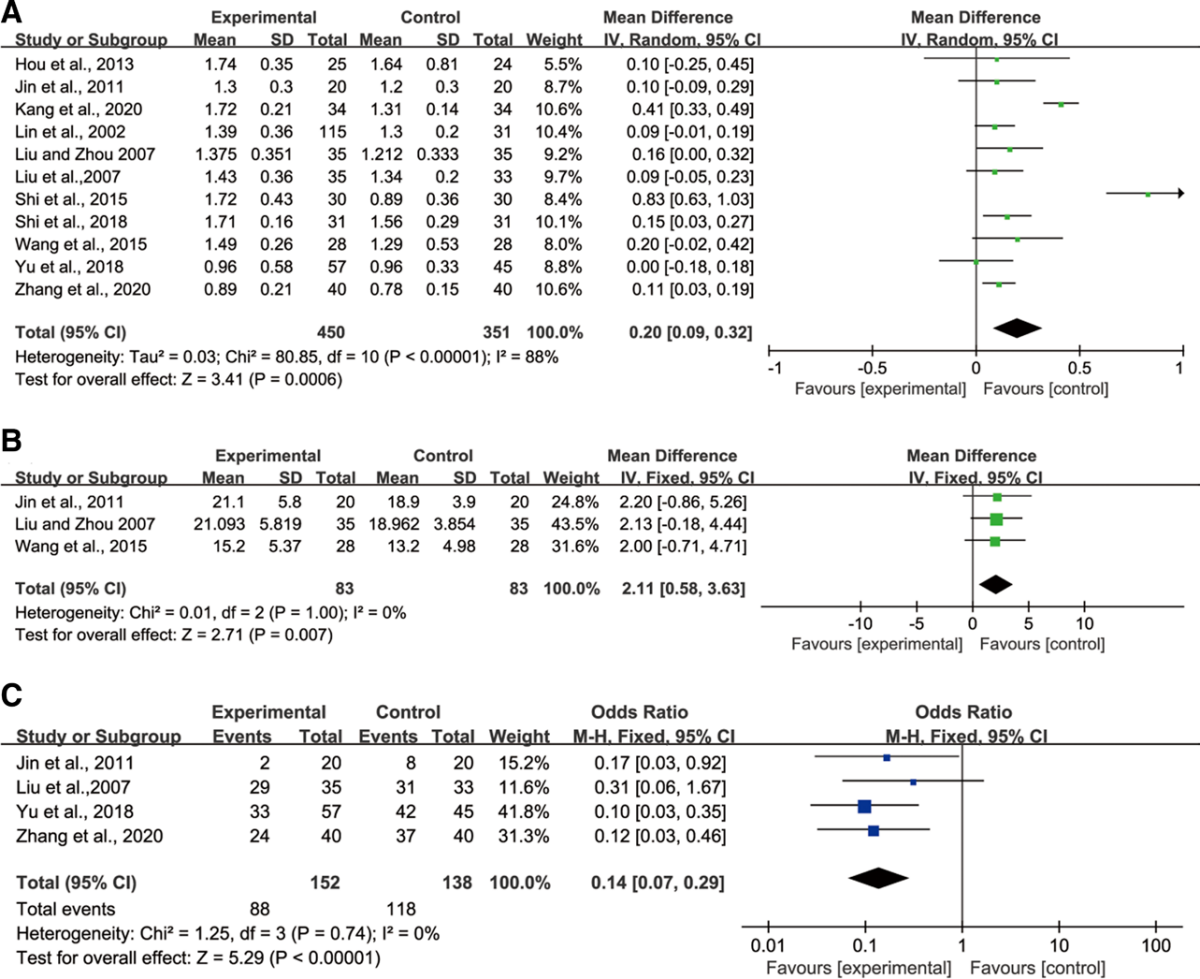
Forest plot (A) CD4^+^/CD8^+^T lymphocytes in Peripheral Blood, (B) NK cells in Peripheral Blood, and (C) leukopenia in Peripheral Blood. NK = natural killer cell.

Eleven studies reported the effect of ginsenoside Rg3 combined with chemotherapy and conventional chemotherapy on the ratio of CD4^+^/CD8^+^ T lymphocytes in peripheral blood of patients, including 450 patients in the experimental group and 351 patients in the control group. The results of the heterogeneity test (*P* < .00001, *I*^2^ = 88%) were shown in Figure 4A. The results demonstrated that the ginsenoside Rg3 in combination with the chemotherapy group was statistically effective in increasing the CD4^+^/CD8^+^ ratio in peripheral blood (MD = 0.20; 95% CI: 0.09, 0.32; *P* = .0006). Subgroup analysis on the basis of treatment cycle indicated that ≤ 8 weeks (MD = 0.20; 95% CI: 0.15, 0.25; *P* < .00001) was more effective in elevating the CD4^+^/CD8^+^ ratio than > 8 weeks (MD = 0.19; 95% CI: 0.13, 0.25; *P* < .00001) (Figure S3, Supplemental Digital Content, http://links.lww.com/MD/I760).

#### 3.4.5. NK cell.

Three included studies were conducted to determine changes in the activity of NK cells in the experimental group (83 patients) and control groups (83 patients). Figure 4B illustrates that there was no heterogeneity (*P* = 1.00, *I*^2^ = 0%) among the studies, and a fixed-effect model could be used for Meta-analysis. The ginsenoside Rg3 combined with the chemotherapy group effectively increased the activity of NK cell levels with a statistically significant difference (MD = 2.11; 95% CI: 0.58, 3.63; *P* = .007).

#### 3.4.6. WBC.

The decline of WBC after administration of ginsenoside Rg3 in combination with chemotherapy versus regular chemotherapy was reported in 4 studies, with152 patients in the test group and 118 patients in the control group. The results of the heterogeneity test (*P* = .74, *I*^2^ = 0%) were shown in Figure 4 C. The results showed that the decline of WBC in the experiment group was lower than in the chemotherapy group, which could effectively reduce the adverse effects of leukocytopenia brought by chemotherapy (MD = 0.14; 95% CI: 0.07, 0.29; *P* < .00001).

#### 3.4.7. Clinical efficacy.

**Figure 5.**
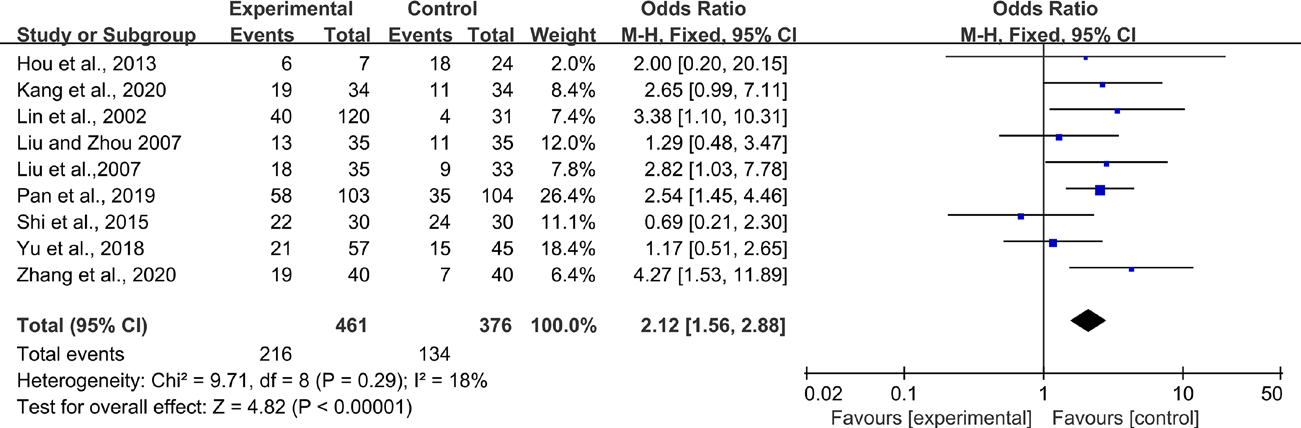
Forest plot of the randomized controlled trials investigating clinical efficacy in Peripheral Blood in NSCLC patients.

The clinical efficacy in the ginsenoside Rg3 combined with the chemotherapy group and conventional chemotherapy group were studied in 9 RCTs, including 461 patients in the experimental group and 376 patients in the control group. The results of the heterogeneity test (*P* = .29, *I*^2^ = 18%) were shown in Figure 5. The heterogeneity among studies was <50%, and a fixed-effects model was used for Meta-analysis. The results showed that the clinical efficacy of the ginsenoside Rg3 combined chemotherapy group was higher than that of the chemotherapy alone group, which could significantly improve the clinical efficacy for patients with statistically significant differences (MD = 2.12; 95% CI: 1.56, 2.88; *P* < .00001).

### 3.5. Publication bias assessment

**Figure 6.**
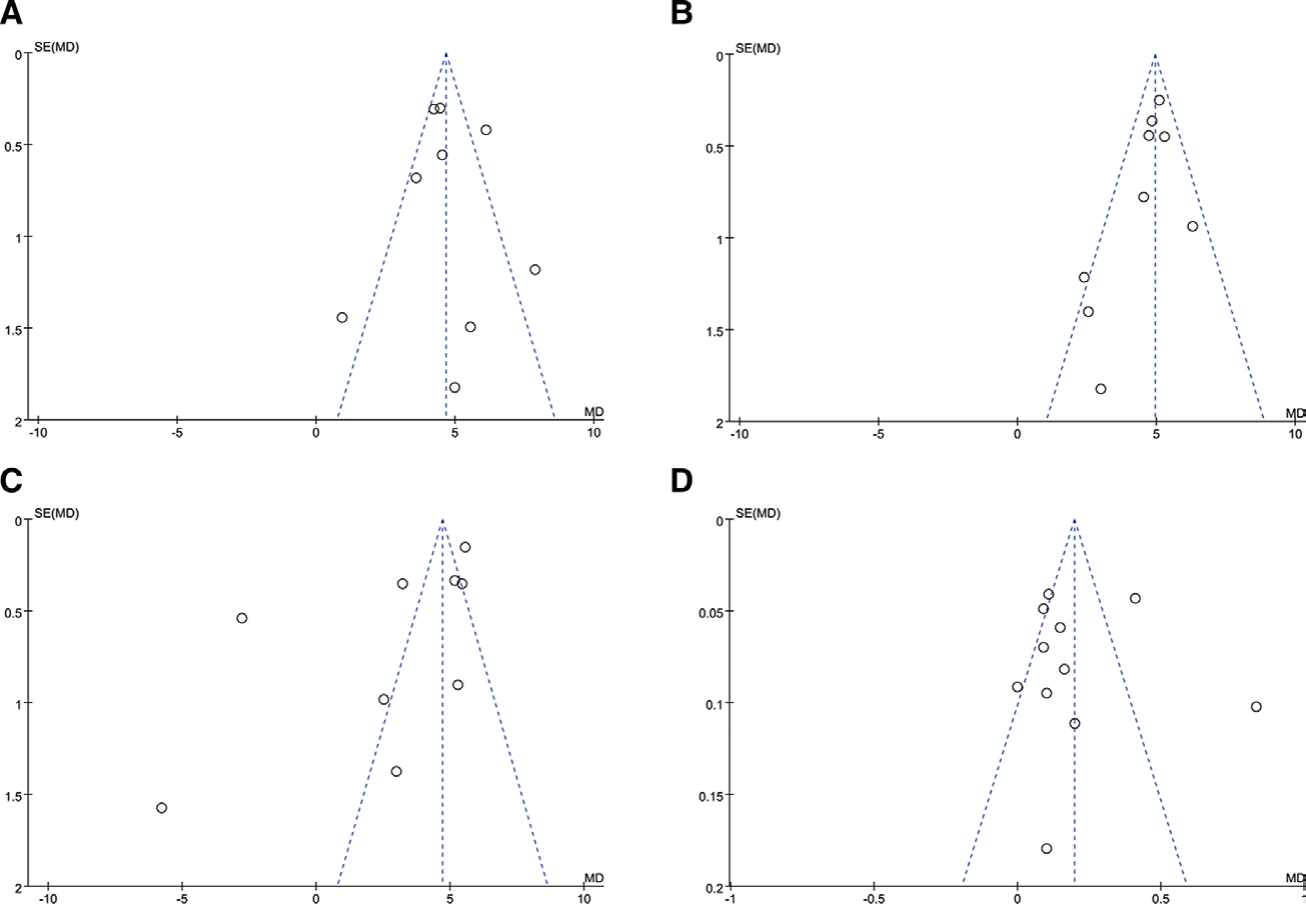
Funnel plot of the mean difference (MD) versus the S.E. of the weighted mean difference (MD).

Funnel plots were drawn to assess the presence of slight sample size publication bias (Fig. 6). The results revealed that the included studies were generally distributed on both sides of the middle line of the funnel plot, suggesting a low risk of publication bias. Some studies deviated from the midline close to the X-axis, suggesting a possible small sample size study effect. The funnel plots of CD3^+^ (Fig. 6A and B) and CD4^+^ levels were generally symmetrical, while the funnel plots of CD8^+^ and CD4^+^/CD8^+^ ratios (Fig. 6C and D) were scattered in some studies. The funnel plot shows that the scatter distribution was asymmetric and skewed, which indicates that there might be publication bias or a small sample effect.

### 3.6. Sensitivity analysis

**Figure 7.**
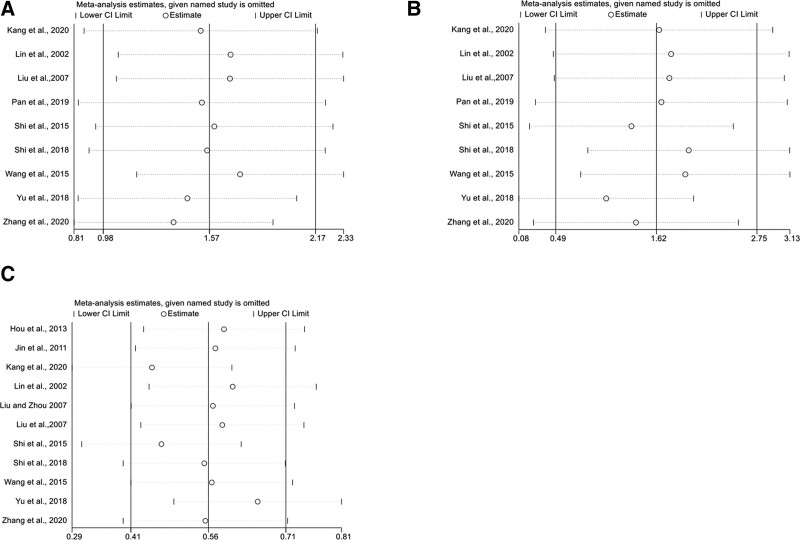
Sensitivity analysis plots.

To visually determine the differences between studies and observe the impact of individual studies on the overall results, sensitivity analysis was performed to determine the source of heterogeneity among the included studies (Fig. 7). For the outcome of CD3^+^ T cell, the heterogeneity was significantly reduced after excluding the 2 papers (*P* = .09, *I*^2^ = 46%) during sensitive analysis.^[19,29]^ The heterogeneity of the CD8^+^ level had no significant changes after excluding the RCTs of literature (*P* < .00001, *I*^2^ = 97%).^[19]^ For the CD4^+^/CD8^+^ ratio, the heterogeneity was significantly reduced after excluding the 2 papers (*P* = .92, *I*^2^ = 0%).^[24,25]^

## 4. Discussions

Most chemotherapeutic drugs used to treat malignant tumors are highly cytotoxic. Due to the poor selectivity, while killing tumor cells, they also have toxic side effects on some normal cells, including immune cells, leading to a decrease in the immune function of patients.^[31]^ Meanwhile, various cytokines released by the tumor will also affect the immune response of autologous immune cells. Therefore, it is a research challenge to improve the immune function of patients while maintaining the effectiveness of chemotherapy drugs.^[32]^ According to the theory of Chinese medicine, ginseng can intensely replenish vitality, which is consistent with the strengthening of immunity in modern medicine. Ginsenoside Rg3, the main component of ginseng, can improve the antitumor effect by up-regulating the auto-antigenicity and immunogenicity of tumor cells, enhancing the function of immune effector cells and immune-active molecules, and regulating the local immune microenvironment.^[33–35]^ Clinical studies have demonstrated that ginsenoside Rg3 can reduce PD-L1 expression, restore T-cell cytotoxicity against tumors and prolong patient survival by inhibiting nuclear transcription factor-κBp65 (NF-κBp65) and protein kinase B (Akt).^[36,37]^ Zhang et al found that ginsenoside Rg3 combined with chemotherapy increased the levels of NKG2D, γ-interferon (IFN-γ), IL-2 levels, and T-lymphocyte subpopulation in peripheral blood of patients with intermediate to advanced non-small cell lung cancer, thereby improving the immune function of the body.^[38]^ Previous studies have shown that ginsenoside Rg3 can enhance immunity in patients, but statistical evidence is lacking. To assess whether ginsenoside Rg3 in combination with chemotherapy is effective in improving patient immunity, we conducted a comprehensive systematic evaluation of 12 randomized controlled trials in a total of 1008 patients with NSCLC.

T lymphocytes are the critical components in the adaptive immune response. They can bind specifically to target cells to directly destroy or release lymphokines to amplify the immune response involved in the body’s immune response.^[39]^ CD3^+^ cell represents all T cells, and CD4^+^ cell is helper/inducer T cells that produce a large number of cytokines to enhance tumor immune effect after activation. CD8^+^ cell is cytotoxic/suppressive T cells with viral clearance and adhesion function, and the ratio of CD4^+^/CD8^+^ represents the body’s immune status. The lower the value, the poorer the immune response capability of the cells against the tumor.^[40]^ Tumor patients are often in a state of immune imbalance, with reduced CD4^+^ T cells, decreased CD4^+^/CD8^+^, and cellular immune dysfunction.^[41]^ In this study, we found that ginsenoside Rg3 combined with chemotherapy increased the levels of CD3^+^, CD4^+^, CD8^+^, CD4^+^/CD8^+^ in peripheral blood T lymphocyte subsets in NSCLC patients compared with chemotherapy alone. A subgroup analysis of CD3^+^, CD8^+^, and CD4^+^/CD8^+^ T lymphocytes was also performed, and the immune effect of T lymphocytes was improved more with a dosing time of ≤ 8 weeks than with > 8 weeks. The reason for this result might be that the study was conducted with ginsenoside Rg3 in combination with chemotherapy for NSCLC, and the longer the duration of action of the chemotherapy drug, the greater the negative impact on the immune function of the body. Ginsenoside Rg3 can stimulate the expression of CD4^+^ in lymphocytes, increase the activity of CD4^+^ T lymphocytes, promote the secretion of IFN-γ, tumor necrosis factor-β, IL-2, and other antitumor cytokines, upregulate CD4^+^ T/CD8^+^ T ratio, and enhance the immune function of the body.^[42]^

**Figure 8.**
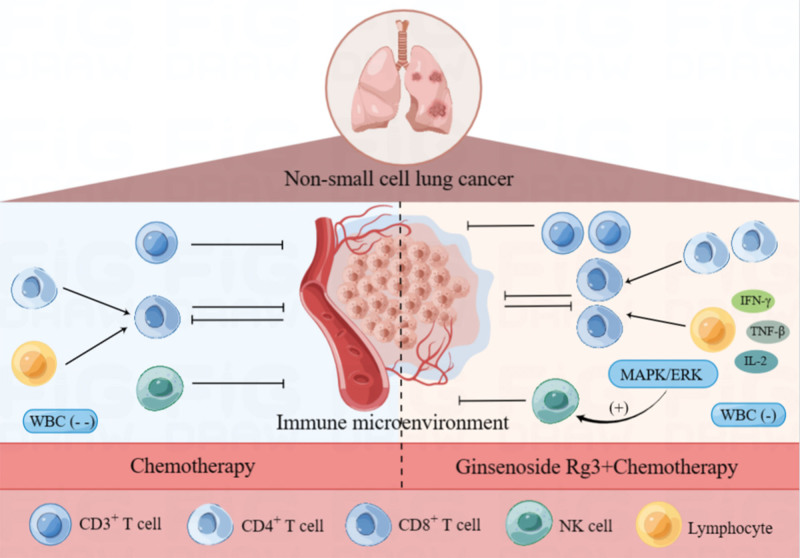
Comparison of the mechanisms of ginsenoside Rg3 combined with chemotherapy and chemotherapy alone on the immune microenvironment of NSCLC. NSCLC = non-small cell lung cancer.

NK cell is the first-line of defense of the body against tumors, and when they are activated by cytokines such as IL-2 and IFN-γ, their roles in killing tumor cells are significantly increased.^[43]^ Our meta-analysis showed that ginsenoside Rg3 combined with chemotherapy could enhance NK cell activity in NSCLC patients compared with the chemotherapy group. It has been documented that ginsenoside Rg3 can improve the activity of natural killer cells by increasing the expression of activated receptors through MAPK/ERK signaling pathway, thereby increasing the binding rate of NK cells and cancer cells, thus improving the immune function of patients and exerting a more powerful antitumor effect^[44]^ (Fig. 8).

Leukocytopenia is a common adverse effect after chemotherapy due to the lack of targeted recognition of tumor cells by chemotherapeutic agents.^[45]^ Patients with decreased WBC cause secondary infections, which in turn prolong the chemotherapy cycle and reduce the effectiveness of chemotherapy.^[46]^ Our studies have found that ginsenoside Rg3 combined with chemotherapy could promote the recovery of leukocytes, reduce the incidence of infection, and reduce the toxicity of chemotherapy in NSCLC patients.^[47]^

Sensitivity analyses were performed to investigate the differences between studies and to observe the impact of individual studies on the overall results. This study showed that individual studies had heterogeneous effects, for outcomes such as the level of CD3^+^ T Lymphocytes and the ratio of CD4^+^/CD8^+^ T Lymphocytes, while for CD8^+^ T lymphocyte level, heterogeneity was not significantly affected by individual studies. Differences in RCTs design, such as the use of multiple chemotherapies such as GP, NP, CP, and TP, may have contributed to heterogeneity. Also, the different acceptance of herbal medicines by patients may have affected the trial effect. The sample sizes of both CD3^+^ and CD8^+^ were <10, and heterogeneity due to the insufficient number of included trials cannot be ruled out. Random-effects models were used in the effects of the above outcomes. However, the results tend to be referential and need further confirmed by more high-quality clinical studies.

The current study still had the following limitations: Traditional Chinese Medicine has been used for thousands of years in eastern countries for its ability to inhibit tumor growth and metastasis, improve antitumor immunity, and reduce the side effects of chemotherapy. However, there might be geographical bias of ginsenoside Rg3 because it was mainly used in China and less in other countries; There may be implementation bias in the studies, mainly due to the small number of included studies, the small number of foreign literatures, and the lack of mention of random assignment plan and blinded evaluation in some studies; The study did not cover all stages of NSCLC, which might result in implementation bias in the studies; Although the dosing guidelines recommend 20 mg of ginsenoside Rg3 orally every time, it is speculated that different dosage will produce implementation bias.

## 5. Conclusion

This systematic evaluation, despite some limitations, provided evidence that ginsenoside Rg3 combined with chemotherapy could improve patients immunity. This study confirmed that ginsenoside Rg3 could inhibit the decrease of the levels of CD3^+^, CD4^+^, CD8^+^, CD4^+^/CD8^+^ T lymphocytes and WBC in NSCLC patients with different degrees, and improve the activity of NK cells, indicating that it could enhance the immune function of patients, reduce the incidence of leukocytopenia after chemotherapy, providing evidence-based evidence for the clinical use of the drug.

## Acknowledgements

This project was funded by the National Natural Science Foundation of China (No.81973488, No.22003002); Natural Science Foundation of Anhui Province (2108085QH371); Key Project of Natural Science Foundation for the Higher Education Institutions of Anhui Province (KJ2019A0451, GXXT-2020-025 (10-4), KJ2020A0382); School Level Student Innovation and Entrepreneurship Training Program of Anhui University of Chinese Medicine (S2022265). Some of the figures in the manuscript were drawn in Figdraw.

## Author contributions

**Conceptualization:** Song Gao, Tiancheng Wang, Nan Wang, Lingfeng Sun, Rongfeng Hu.

**Data curation:** Song Gao, Cancan Fang, Tiancheng Wang, Wenjie Lu, Yang Chen, Rongfeng Hu.

**Formal analysis:** Song Gao, Cancan Fang, Tiancheng Wang, Wenjie Lu, Rongfeng Hu.

**Funding acquisition:** Song Gao, Tiancheng Wang, Rongfeng Hu.

**Investigation:** Song Gao, Cancan Fang, Wenjie Lu, Rongfeng Hu.

**Methodology:** Song Gao, Wenjie Lu, Nan Wang, Wenyou Fang, Rongfeng Hu.

**Project administration:** Song Gao, Wenjie Lu, Lingfeng Sun, Rongfeng Hu.

**Resources:** Song Gao, Cancan Fang, Tiancheng Wang, Wenjie Lu, Rongfeng Hu.

**Software:** Song Gao, Wenjie Lu, Rongfeng Hu.

**Supervision:** Tiancheng Wang, Wenjie Lu, Yang Chen, Rongfeng Hu.

**Validation:** Song Gao, Cancan Fang, Rongfeng Hu.

**Visualization:** Song Gao, Cancan Fang, Wenjie Lu, Nan Wang, Lingfeng Sun, Rongfeng Hu.

**Writing – original draft:** Song Gao, Tiancheng Wang.

**Writing – review & editing:** Yang Chen, Rongfeng Hu.

## Supplementary Material
