# Supplementary material for: The effect of ginsenoside Rg3 combined with chemotherapy on immune function in non-small cell lung cancer: A systematic review and meta-analysis of randomized controlled trials
Source: Medicine (Baltimore). 2022 Apr 7;102(14):e33463. doi: 10.1097/MD.0000000000033463 (PMC10082263; doi:10.1097/MD.0000000000033463)

Figure S1. Forest plot for subgroup analysis of CD_3^+ T lymphocytes in Peripheral Blood in NSCLC patients.

	Experimental			Control				Mean Difference
Study or Subgroup	Mean	SD	Total	Mean	SD	Total	Weight	IV, Fixed, 95% CI
**1.2.1 Dosing time<=8weeks**								
Kang et al., 2020	66.01	2.62	34	61.46	1.91	34	9.4%	4.55 [3.46, 5.64]
Lin et al., 2002	59.67	8.72	120	54.12	7.05	31	1.3%	5.55 [2.62, 8.48]
Liu et al.,2007	59	8	35	54	7	33	0.9%	5.00 [1.43, 8.57]
Wang et al., 2015	69.73	4.13	28	68.8	6.43	28	1.4%	0.93 [-1.90, 3.76]
Yu et al., 2018	7.42	3.18	57	1.31	0.27	45	16.3%	6.11 [5.28, 6.94]
**Subtotal (95% CI)**			**274**			**171**	**29.3%**	**5.30 [4.68, 5.92]**
Heterogeneity: Chi² = 14.69, df = 4 (P = 0.005); I² = 73%								
Test for overall effect: Z = 16.81 (P < 0.00001)								
**1.2.2 Dosing time>8weeks**								
Pan et al., 2019	5.36	2.85	103	1.12	1.3	104	30.7%	4.24 [3.64, 4.84]
Shi et al., 2015	5.03	3.72	30	1.43	0.35	30	6.3%	3.60 [2.26, 4.94]
Shi et al., 2018	62.63	5.13	31	54.77	4.11	31	2.1%	7.86 [5.55, 10.17]
Zhang et al., 2020	6.23	1.85	40	1.77	0.51	40	31.7%	4.46 [3.87, 5.05]
**Subtotal (95% CI)**			**204**			**205**	**70.7%**	**4.39 [3.99, 4.79]**
Heterogeneity: Chi² = 10.27, df = 3 (P = 0.02); I² = 71%								
Test for overall effect: Z = 21.61 (P < 0.00001)								
**Total (95% CI)**			**478**			**376**	**100.0%**	**4.66 [4.32, 4.99]**
Heterogeneity: Chi² = 30.89, df = 8 (P = 0.0001); I² = 74%								
Test for overall effect: Z = 27.27 (P < 0.00001)								
Test for subgroup differences: Chi² = 5.93, df = 1 (P = 0.01), I² = 83.1%								

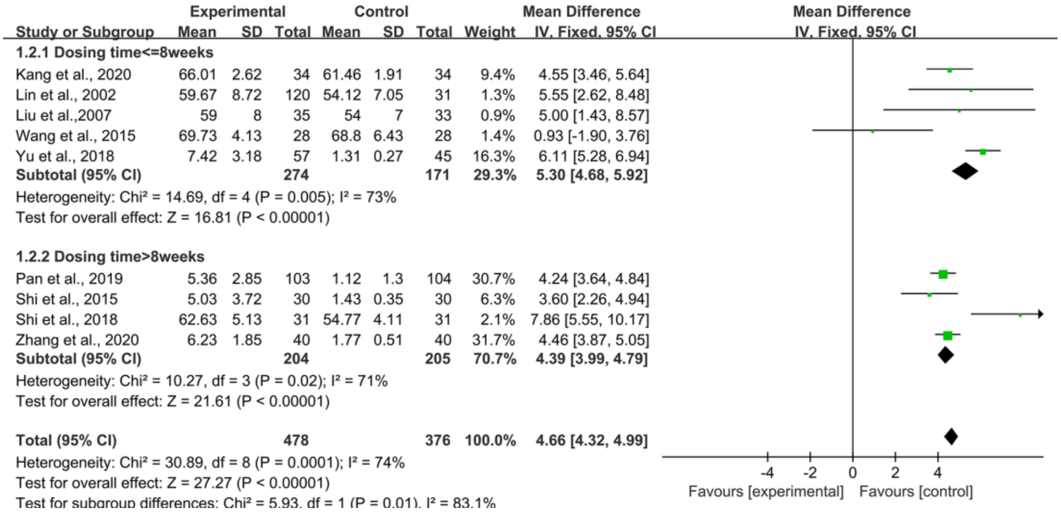

Supplement: Supplementary file 1 [file medi-102-e33463-s001.pdf]
