# Supplementary material for: The effect of ginsenoside Rg3 combined with chemotherapy on immune function in non-small cell lung cancer: A systematic review and meta-analysis of randomized controlled trials
Source: Medicine (Baltimore). 2022 Apr 7;102(14):e33463. doi: 10.1097/MD.0000000000033463 (PMC10082263; doi:10.1097/MD.0000000000033463)

Figure S2. Forest plot for subgroup analysis of CD_8^+ T lymphocytes in Peripheral Blood in NSCLC patients.

Study or Subgroup	Experimental Mean	Experimental SD	Experimental Total	Control Mean	Control SD	Control Total	Weight	Mean Difference IV, Fixed, 95% CI
**3.2.1 Dosing time<=8weeks**								
Kang et al., 2020	38.52	3.81	34	33.24	3.62	34	1.7%	5.28 [3.51, 7.05]
Lin et al., 2002	28.47	7.15	115	25.93	4.01	31	1.4%	2.54 [0.62, 4.46]
Liu et al.,2007	28	7	35	25	4	33	0.7%	3.00 [0.31, 5.69]
Wang et al., 2015	22.68	6.16	28	28.42	5.61	28	0.6%	-5.74 [-8.83, -2.65]
Yu et al., 2018	7.05	1.16	57	1.51	0.22	45	56.4%	5.54 [5.23, 5.85]
**Subtotal (95% CI)**			**269**			**171**	**60.8%**	**5.33 [5.03, 5.62]**
Heterogeneity: Chi² = 62.18, df = 4 (P < 0.00001); I² = 94%								
Test for overall effect: Z = 35.22 (P < 0.00001)								
**3.2.2 Dosing time>8weeks**								
Pan et al., 2019	6.34	3.46	103	3.11	0.87	104	11.3%	3.23 [2.54, 3.92]
Shi et al., 2015	6.87	1.83	30	1.71	0.34	30	12.0%	5.16 [4.49, 5.83]
Shi et al., 2018	18.21	2.02	31	20.98	2.22	31	4.8%	-2.77 [-3.83, -1.71]
Zhang et al., 2020	7.31	2.16	40	1.85	0.59	40	11.1%	5.46 [4.77, 6.15]
**Subtotal (95% CI)**			**204**			**205**	**39.2%**	**3.72 [3.35, 4.09]**
Heterogeneity: Chi² = 188.99, df = 3 (P < 0.00001); I² = 98%								
Test for overall effect: Z = 19.76 (P < 0.00001)								
**Total (95% CI)**			**473**			**376**	**100.0%**	**4.70 [4.47, 4.93]**
Heterogeneity: Chi² = 295.29, df = 8 (P < 0.00001); I² = 97%								
Test for overall effect: Z = 39.83 (P < 0.00001)								
Test for subgroup differences: Chi² = 44.12, df = 1 (P < 0.00001), I² = 97.7%								

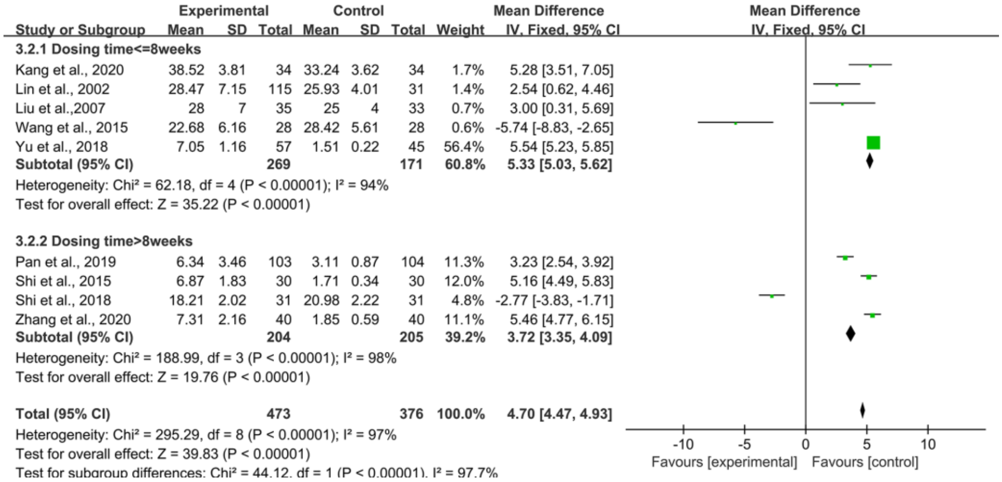

Supplement: Supplementary file 2 [file medi-102-e33463-s002.pdf]
