# Supplementary material for: The effect of ginsenoside Rg3 combined with chemotherapy on immune function in non-small cell lung cancer: A systematic review and meta-analysis of randomized controlled trials
Source: Medicine (Baltimore). 2022 Apr 7;102(14):e33463. doi: 10.1097/MD.0000000000033463 (PMC10082263; doi:10.1097/MD.0000000000033463)

Figure S3. Forest plot for subgroup analysis of $CD4^{+}/CD8^{+}$ T lymphocytes in Peripheral Blood in NSCLC patients.

	Experimental			Control				Mean Difference
Study or Subgroup	Mean	SD	Total	Mean	SD	Total	Weight	IV, Fixed, 95% CI
**4.2.1 Dosing time<=8wweeks**								
Hou et al., 2013	1.74	0.35	25	1.64	0.81	24	1.2%	0.10 [-0.25, 0.45]
Jin et al., 2011	1.3	0.3	20	1.2	0.3	20	4.2%	0.10 [-0.09, 0.29]
Kang et al., 2020	1.72	0.21	34	1.31	0.14	34	20.4%	0.41 [0.33, 0.49]
Lin et al., 2002	1.39	0.36	115	1.3	0.2	31	15.8%	0.09 [-0.01, 0.19]
Liu and Zhou 2007	1.375	0.351	35	1.212	0.333	35	5.7%	0.16 [0.00, 0.32]
Liu et al.,2007	1.43	0.36	35	1.34	0.2	33	7.8%	0.09 [-0.05, 0.23]
Wang et al., 2015	1.49	0.26	28	1.29	0.53	28	3.1%	0.20 [-0.02, 0.42]
Yu et al., 2018	0.96	0.58	57	0.96	0.33	45	4.6%	0.00 [-0.18, 0.18]
**Subtotal (95% CI)**			**349**			**250**	**62.7%**	**0.20 [0.15, 0.25]**
Heterogeneity: Chi² = 37.44, df = 7 (P < 0.00001); I² = 81%								
Test for overall effect: Z = 8.12 (P < 0.00001)								
**4.2.2 Dosing time>8weeks**								
Shi et al., 2015	1.72	0.43	30	0.89	0.36	30	3.6%	0.83 [0.63, 1.03]
Shi et al., 2018	1.71	0.16	31	1.56	0.29	31	10.8%	0.15 [0.03, 0.27]
Zhang et al., 2020	0.89	0.21	40	0.78	0.15	40	22.9%	0.11 [0.03, 0.19]
**Subtotal (95% CI)**			**101**			**101**	**37.3%**	**0.19 [0.13, 0.25]**
Heterogeneity: Chi² = 43.36, df = 2 (P < 0.00001); I² = 95%								
Test for overall effect: Z = 6.00 (P < 0.00001)								
**Total (95% CI)**			**450**			**351**	**100.0%**	**0.20 [0.16, 0.24]**
Heterogeneity: Chi² = 80.85, df = 10 (P < 0.00001); I² = 88%								
Test for overall effect: Z = 10.09 (P < 0.00001)								
Test for subgroup differences: Chi² = 0.04, df = 1 (P = 0.83), I² = 0%								

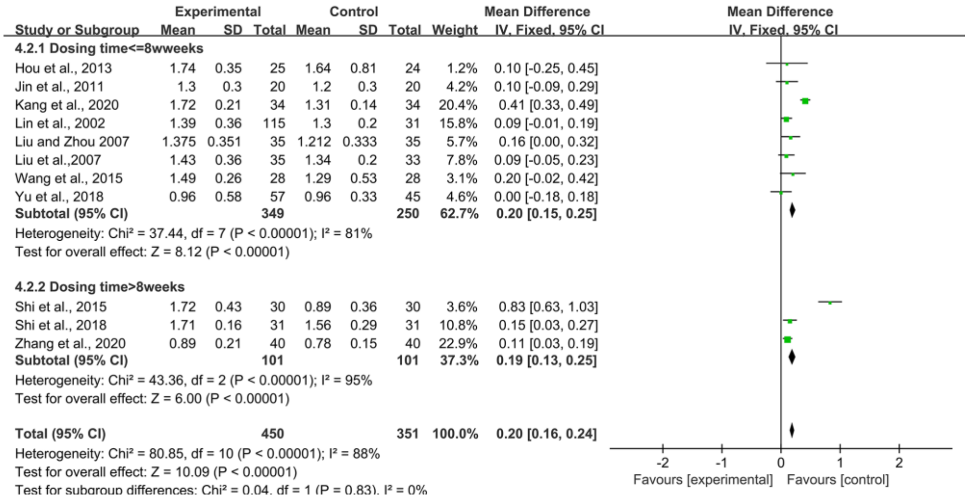

Supplement: Supplementary file 3 [file medi-102-e33463-s003.pdf]
